# Sinonasal NUT-Midline Carcinoma – A Multimodality Approach to Diagnosis, Staging and Post-Surgical Restaging

**DOI:** 10.7759/cureus.288

**Published:** 2015-07-26

**Authors:** Faiq Shaikh, Nitin Pagedar, Omer Awan, Parren McNeely

**Affiliations:** 1 Imaging Informatics, University of Pittsburgh Medical Center; 2 Molecular Imaging Physician, S&L Readings, LLC.; 3 CEO, Crunchtimr Medical Solutions, LLC; 4 Otolaryngology - Head and Neck Surgery, University of Iowa Hospitals & Clinics, Iowa City, IA; 5 Department of Radiology, Dartmouth Hitchcock Medical Center; 6 Department of Radiology, University of Iowa Hospitals & Clinics, Iowa City, IA

**Keywords:** nut, midline carcinoma, multimodality, mri, pet-ct, sinonasal

## Abstract

Nuclear protein testis (NUT) midline carcinoma is a rare malignancy involving predominantly the midline structures of the body. It is characterized by its genotypic feature of BRD4-NUT translocation, which is in contrast with other malignant processes that are usually categorized based on their histologic/phenotypic features. As these tumors may vary in their histologic presentation, they can be misdiagnosed as poorly differentiated carcinomas. Moreover, they are often very aggressive and associated with high mortality. Therefore, it is extremely important to diagnose them early using computed tomography (CT) and magnetic resonance imaging (MRI) and perform staging and restaging using 18-fluorodeoxyglucose positron emission tomography/computed tomography (18-FDG PET/CT), in addition to accurately identifying them at a microscopic and molecular level. We report a unique case of a sinonasal NUT midline carcinoma that was diagnosed with CT, staged with PET/CT, and restaged using PET/CT and MRI.

## Introduction

We present here a rare case of nuclear protein testis (NUT) midline carcinoma involving sinonasal structures. This is an extremely aggressive malignant tumor; its management involved a multimodality approach comprising of computed tomography (CT) for diagnosis and 18-fluorodeoxyglucose positron emission tomography/computed tomography (18F-FDG PET/CT) as well as magnetic resonance imaging (MRI) for staging and restaging.

## Case presentation

A 29-year-old female presented with excessive tearing of the left eye in August 2014. She had also noticed headaches originating on the left side of the face and pain under the left eye, as well as fatigue and left submandibular swelling over the past several months. Informed patient consent was obtained prior to her treatment. 

**Figure 1:**
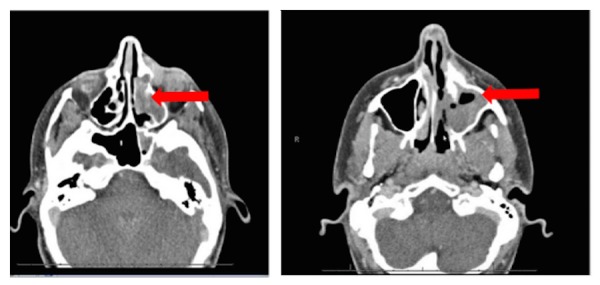
Presurgical CT Presurgical contrast-enhanced axial CT images of the head demonstrate an infiltrative mass in the left medial canthus region adjacent to the nasolacrimal duct, with resulting effacement of the left inferior meatus and partial effacement of the left middle meatus (see arrow). Also noted was adjacent hyperostosis along the anteromedial left maxillary sinus and additional involvement of the nasal cavity (see arrow).

She underwent her first CT of the maxillofacial structures in October, 2014, which revealed a 1.5 x 0.9 x 1.5 cm mass in the left medial canthus region adjacent to the nasolacrimal duct with associated effacement of the left inferior meatus and partial effacement of the left middle meatus. Also noted was periosteal reaction along the left medial maxillary sinus wall with adjacent hyperostosis along the anteromedial left maxillary sinus (Figure 1). She was taken to the operating room for endoscopic sinus surgery, during which a subtotal resection of the mass was undertaken. The pathology report showed squamous cell carcinoma.

**Figure 2:**
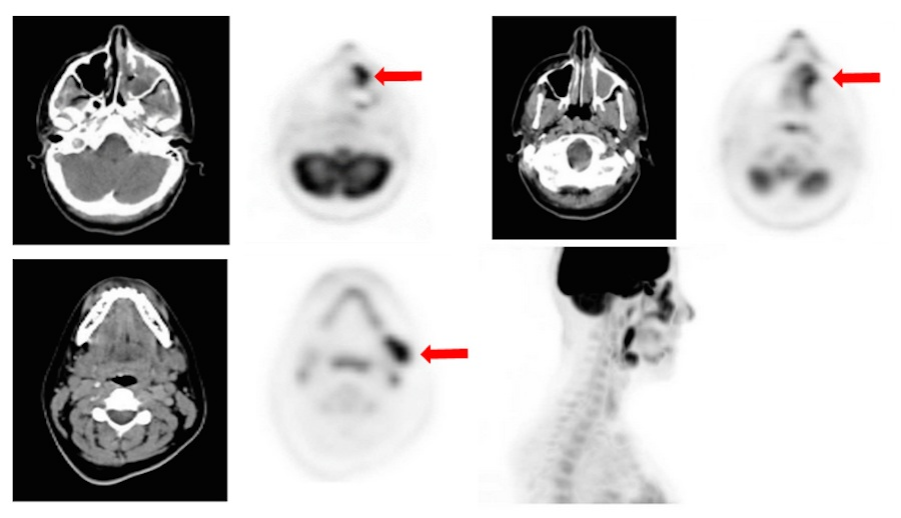
Staging FDG-PET/CT PET/CT images reveal increased FDG avidity within the left medial canthus and left nasal cavity soft tissues and a left cervical level IB lymph node (see arrows).​

The following staging CT of the chest, abdomen, and pelvis revealed no evidence of pulmonary nodules, hilar/mediastinal lymphadenopathy, visceral or osseous metastatic disease. A staging PET/CT scan was then performed that showed increased FDG avidity within the soft tissue mass involving the left medial canthus and the left nasal cavity as well as hypermetabolic activity in bilateral jugulodigastric lymph nodes and slightly smaller lymph nodes deep to the sternocleidomastoid muscles (Figure 2). A hypermetabolic lymph node measuring 2.8 x 1.7 cm was seen anterior to the left submandibular gland. Additional FDG avidity was seen in subcentimeter nodes along the base of the neck bilaterally. There was no evidence of distant metastatic disease. She also underwent an MRI face/sinus, which revealed a residual T2 hypointense, enhancing soft tissue mass involving the anterior left maxillary sinus, premaxillary soft tissues, and extending superiorly into the extraconal soft tissues of the left orbit.

Bilateral enhancing Level IB and Level IIB adenopathy was also present. Based on the above clinical picture, she was returned to the OR for a left medial maxillectomy with dissection of the intraorbital tumor performed through an open approach, along with bilateral neck dissection. Pathology confirmed poorly differentiated squamous cell carcinoma; P16 was partially positive and EBER was negative. There were multiple involved margins in the orbit as well as at the inferior maxillary bone. The pathology was sent for review to another hospital for a second review, where it was confirmed as a rare midline NUT carcinoma.

**Figure 3:**
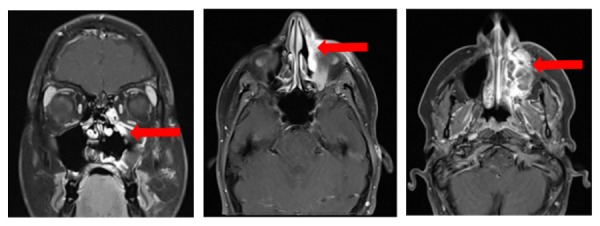
Postsurgical MRI Gadolinium contrast-enhanced T2-weighted coronal and axial MRI images demonstrate a residual T2 hypointense, enhancing soft tissue mass involving the anterior left maxillary sinus, premaxillary soft tissues, and extending​ superiorly into the extraconal soft tissues of the left orbit (see arrows).​

**Figure 4:**
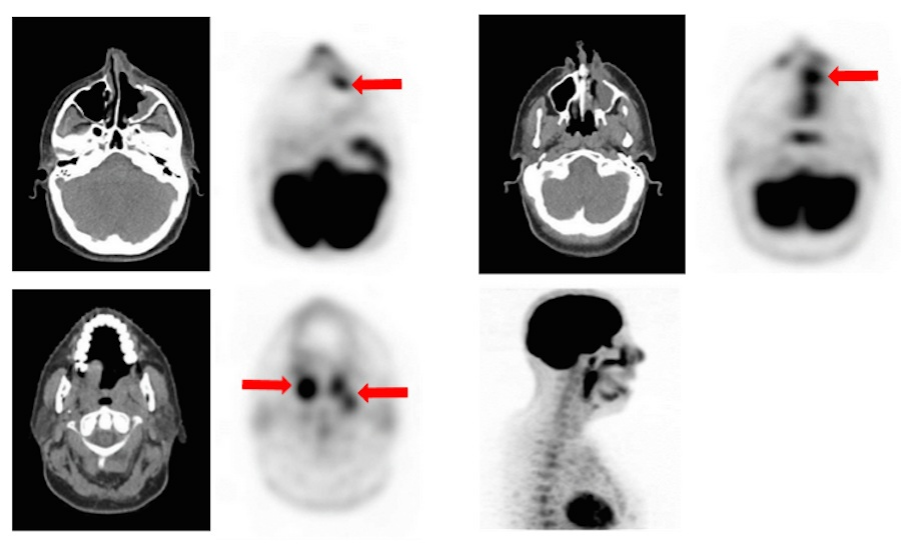
Restaging FDG-PET/CT PET-CT revealing FDG-avid lesions in the anteromedial left​ maxillary sinus surgical bed, as well as FDG-avid right adenoid tonsillar lesion, squamous temporal bone involvement, and left parapharyngeal nodes (see arrows).

A follow-up post-surgical MRI of the face/sinus showed residual disease involving the posterior margin of the maxillary sinus and interval enlargement of a left retropharyngeal lymph node, concerning for the progression of her disease (Figure 3). PET-CT scan confirmed the persistent residual disease in the left hard palate, anteromedial left maxillary sinus, and left cervical nodal metastases (Figure 4). Therefore, she was started concurrent chemoradiation therapy with high-dose cisplatin, extrapolating from the EORTC22931 and RTOG95-01 trials. Further follow-up on the patient's response to medication was unavailable at the time of this report.

## Discussion

NUT (nuclear protein testis) midline carcinoma is a rare malignancy that is defined by its genotype instead of histologic features. It is characterized by a BRD4-NUT translocation that affects a broad age group (range: 0.1 - 78 years), with similar predisposition among men and women. Adults have been found to have greater than an 80% likelihood of death within the first year after diagnosis [1]. These tumors tend to arise in the midline anatomic locations, most commonly in the upper aero-digestive tract (UADT) (50%) as was the case with our patient, and the mediastinum (41%). Other sites include the parotid gland, pancreas, adrenal gland, orbit, lung, bladder, and iliac bone [2].

Until recently, the diagnosis depended on demonstration of the NUT translocation with conventional karyotyping, reverse transcriptase-polymerase chain reaction, or split apart fluorescence in situ hybridization studies [3]. New advances in immunohistochemistry studies using rabbit monoclonal antibodies raised against amino acids 450 to 700 of the human NUT protein are promising, with a demonstrated 87% sensitivity and 100% specificity, a negative predictive value of 99%, and a positive predictive value of 100% in differentiating NUT midline carcinoma (NMC) from other types of carcinoma [4]. Diagnosis is now made on the identification of a genetic change (a rearrangement involving the NUT locus at 15q14) that generates a specific fusion transcript with a member of the bromodomain-containing protein (BRD) family, such as BRD4 located on chromosome 19p13 [5-6]. In the variant subtype of NMC, an NSD3-NUT fusion gene has been identified, although it has been reportedly amplified in breast cancer and mutated in pancreas and lung cancers [6].

The histologic features of the tumor biopsied from our patient were most consistent with those of a poorly differentiated carcinoma, which are characterized by round, blue, cell tumor morphology. These are generally categorized as sinonasal undifferentiated carcinoma, but could represent NMC based on their genotypic and phenotypic features. NMC is variably positive for a number of IHC stains, including several cytokeratin markers (e.g., CK AE1/AE3, CK5/6, etc.) and IgA-endomysial antibodies (EMA) [6]. It may also express positivity for p16, which was seen with a histologic specimen from our patient's tumor. Nuclear staining with the NUT antibody is not entirely restricted to NMC, and it can be positive in germ cell tumors (<5% of cell nuclei, in a smooth pattern of nuclear staining) in which endogenous NUT is expressed. In undifferentiated neoplasms of the UADT and mediastinum, this is a consideration only when clinical signs or imaging studies support the possibility of a metastatic germ cell tumor [4]. NMCs of the lung have been reported, but they are extremely rare; only five cases, including two pediatric and three adult cases, have been documented [6]. Bishop and Westra found a 2% incidence of NMC in 151 sinonasal tumors [7].

It is suspected that NMC is underdiagnosed, given its histologic crossover with other types of the aforementioned cancers. Evans, et al. found a 3.5% incidence of NMC in 114 cases of poorly differentiated carcinomas or unclassified mediastinal malignancies [8]. Various undifferentiated carcinomas and poorly differentiated carcinomas have been found to have an underlying NMC genotype [9-15]. NMC tumors have been typically identified to have an epithelioid component with an expression of cytokeratin proteins. In contrast to other poorly differentiated carcinomas, the cells comprising NUT midline carcinoma appear rather uniform with round nuclear contours and are medium-sized. In addition, these tumors may have focal squamous differentiation, which is an aggressive variant of squamous cell carcinoma. No single tissue type of origin has been successfully identified for these tumors [16]. It has been recommended to perform immunohistochemical testing for NUT expression in all poorly differentiated carcinomas without glandular differentiation arising in the chest, head, and neck [19].

Clinically, NMC exhibits a locally aggressive behavior, early hematogenous metastasis, and a lack of response to the typical therapeutic regimens in carcinoma treatment [1]. However, more recently with the molecular basis of NMC being more completely understood, more effective treatment options are under investigation, making the early and accurate diagnosis of NMC a priority [5]. As of now, NMC is generally considered to be an incurable tumor with a median overall survival of 6.7 months, and only one known case of NMC has been reported to be successfully treated [1, 10]. In its management, traditional chemotherapeutic and radiotherapeutic regimens may be effective early in the disease, but patients frequently relapse later [6, 9]. In our patient, cisplatin-based chemotherapy was initiated, given the rapid progression proven by MRI and metastatic disease established by PET/CT. Engleson, et al. reported the case of a 30-year-old woman treated with vincristine, ifosfamide, doxorubicin, and etoposide followed by radiation therapy with concurrent weekly docetaxel. Although the patient died suddenly after respiratory failure, the autopsy revealed minimal residual disease [15].

Within the realm of imaging, conventional CT and MRI signal characteristics of NMC are nonspecific and may mimic a number of pathological entities, including lymphoma, metastatic neuroendocrine tumors, and sarcomas [16]. It has been observed to represent imaging features of a hypoattenuating mass demonstrating heterogeneous enhancement with tumor and lymph node necrosis [16, 18, 20-21]. In our patient, FDG PET/CT was helpful in the presurgical staging, which included characterizing the primary lesion, identifying nodal metastasis, excluding distant metastasis, and also played an important role in detecting residual disease postsurgically. There have been reports advocating the use of PET/CT for initial staging as well as for monitoring therapy response assessment [17-18]. Potential pitfalls include low FDG-avidity due to underlying tumoral necrosis from an outgrowth of blood supply. Overall, however, FDG-avidity on PET/CT directly correlated with tumor burden on CT as well as the clinical disease status [21-22].

Contrast-enhanced CT is currently considered the standard of care for initial staging of NMC while MRI plays an adjunctive role and is particularly helpful for addressing concerns for vascular invasion. Additionally, cardiac MRI with dark blood sequences, such as double inversion recovery and HASTE, may offer superior delineation of intracardiac and intravascular tumor extension. Neck MRI may also provide information regarding the presence of bone marrow invasion, perineural involvement, and skull base invasion. This information is critical in radiation therapy treatment planning and helps determine the feasibility of surgical resection. The MRI features of these tumors have been described as heterogeneous with a predominantly hypointense signal on T1- weighted images and a hyperintense signal on T2-weighted images. Contrast-enhanced MRI images show in exquisite detail the marked central necrosis in these lesions and also reveal smaller lesions not appreciated on CT [23]. After a confirmed diagnosis of an NUT midline carcinoma, 18F-FDG PET/CT is the desired modality for the assessment of metastatic disease and for the guidance of biopsying viable tumor tissue. It is also the preferred modality for the assessment of disease response to treatment and aids in the assessment of disease activity over time [23].

## Conclusions

Overall, this case presents a nice example of the complementary roles of FDG-PET/CT, contrast-enhanced CT, and MRI in characterizing NMC disease burden for staging purposes and therapy response monitoring, which is especially crucial for addressing rare and aggressive malignant tumors, such as NMC.
